# Supplementary material for: Endurance Exercise Does Not Exacerbate Cardiac Inflammation in BALB/c Mice Following mRNA COVID-19 Vaccination
Source: Vaccines (Basel). 2024 Aug 26;12(9):966. doi: 10.3390/vaccines12090966 (PMC11435831; doi:10.3390/vaccines12090966)

Endurance exercise does not exacerbate cardiac inflammation in BALB/c mice following mRNA COVID-19 vaccination

Sander Eens, MSc[a,b], Manon Van Hecke, MD[c], Siel Van den Bogaert, PharmD[b], Kasper Favere, MD, PhD[a,b,d,e], Nathalie Cools, MSc, PhD[f], Erik Fransen, MSc, PhD[g], Tania Roskams, MD, PhD[c†], Hein Heidbuchel, MD, PhD[a,d†], Pieter-Jan Guns, PharmD, PhD[b†]

[a]Research Group Cardiovascular Diseases, GENCOR, University of Antwerp, 2610 Antwerp, Belgium
[b]Laboratory of Physiopharmacology, GENCOR, University of Antwerp, 2610 Antwerp, Belgium
[c]Laboratory of Translational Cell and Tissue Research, Department of Imaging and Pathology, University of Leuven, 3000 Leuven, Belgium
[d]Department of Cardiology, Antwerp University Hospital, 2650 Antwerp, Belgium
[e]Department of Internal Medicine, Ghent University, 9000 Ghent, Belgium
[f]Laboratory of Experimental Hematology, Vaccine and Infectious Disease Institute, University of Antwerp, Universiteitsplein 1, B-2610 Antwerp, Belgium
[g]Centre of Medical Genetics, University of Antwerp and Antwerp University Hospital, 2610 and 2650 Antwerp, Belgium

†Shared senior authorship

Correspondence
Sander Eens
Research Group Cardiovascular Diseases, GENCOR
University of Antwerp
Universiteitsplein 1
2610 Antwerp
Belgium
Email: sandereens@uantwerpen.be

Funding
SE is a predoctoral researcher funded by a Research Foundation Flanders (FWO) TBM project (Master@Heart OZ7552). KF is a predoctoral research fellow of the Research Foundation Flanders (FWO) (grant no. 11C6321N). MVH is a predoctoral researcher funded by a Research Foundation Flanders (FWO) project grant (G099222N, to HH, TR and PJG).

Conflict of interest
The authors declare no conflict of interest

Supplementary figure legends and figures

<u>Supplementary Figure 1. Validation cardiac hypertrophy measurement.</u>

The semi-automated mean nuclei count method of our study used to evaluate cardiac hypertrophy of mice was validated by the 'golden standard' mean linear intercept method, showing good correlation between both methods (r=0.735). Pearson's correlation test. Group sizes: SED: n=19, EEX: n=17. Data are expressed as boxplots.

<u>Supplementary Figure 2. Exercise training effects.</u> **A**, Design of the daily treadmill running and treadmill exhaustion testing protocols. **B**, Evaluation of exercise capacity as determined by treadmill exhaustion testing. The Y-axis indicates the difference between both exhaustion tests (week 4 – baseline). No significant differences in exercise capacity were observed between sedentary and exercising mice during week 4 of the exercise intervention (*P*=0.0806). Group sizes: SED: n=41; EEX: n=40. **C**-**D**, Evaluation of exercise-induced cardiac hypertrophy by (**C**) heart-to-body weight ratios and (**D**) cardiomyocyte nuclei counts. Lower nuclei counts correspond to larger cardiomyocyte sizes, suggesting cardiac hypertrophy. At sacrifice, exercising mice showed significantly higher heart-to-body weight ratios (*P*=0.0051) and lower nuclei counts (*P*=0.0010). Group sizes; SED: n=43-44, EEX: n=43-44. Data are represented as boxplots. Individual values are additionally shown. (**B**-**D**) Two-tailed unpaired t-test. EEX: exercise, LV: left ventricular, SED: sedentary, V_{max}: maximal velocity. ** $P < 0.01$, *** $P < 0.001$.

<u>Supplementary Figure 1. Validation cardiac hypertrophy measurement</u>

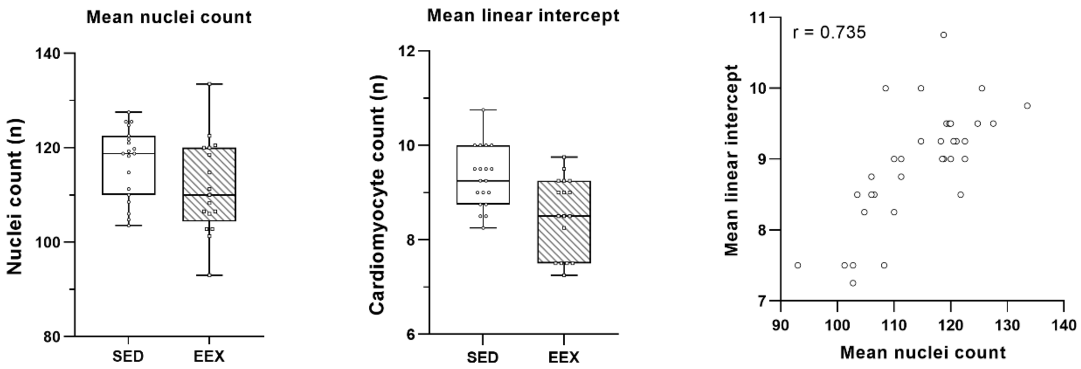

<u>Supplementary Figure 2. Exercise training effects.</u>

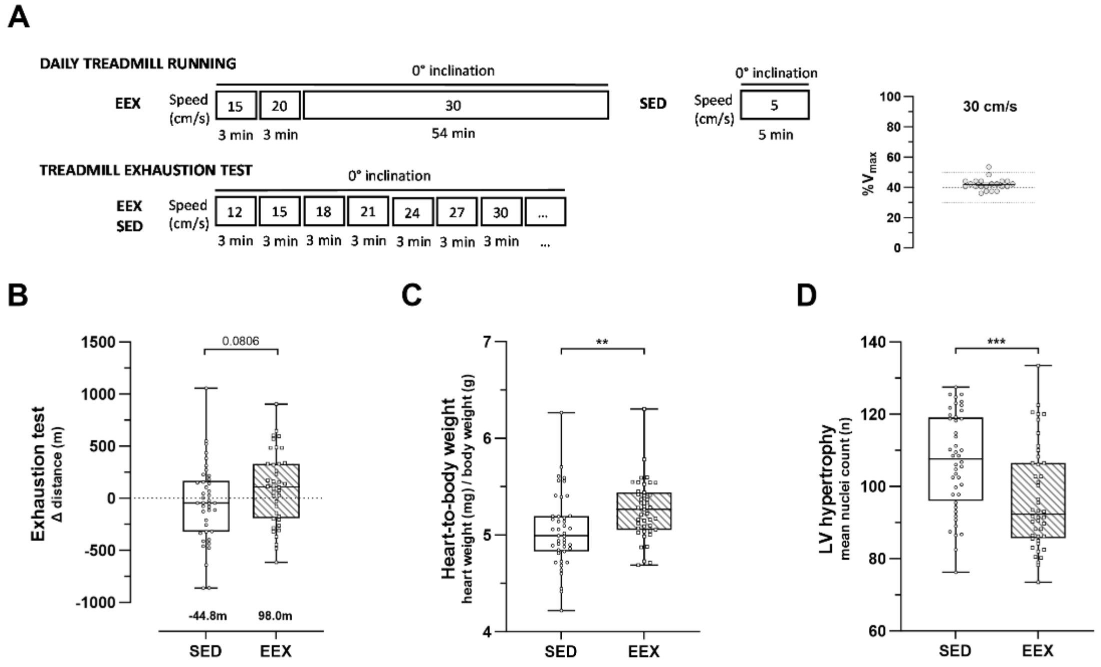

Supplement: Supplementary file 1 [file vaccines-12-00966-s001.zip › vaccines-3134219-supplementary.pdf]
